# Saxitoxin: A Comprehensive Review of Its History, Structure, Toxicology, Biosynthesis, Detection, and Preventive Implications

**DOI:** 10.3390/md23070277

**Published:** 2025-07-02

**Authors:** Huiyun Deng, Xinrui Shang, Hu Zhu, Ning Huang, Lianghua Wang, Mingjuan Sun

**Affiliations:** 1 Department of Student team, College of Basic Medical Sciences, Naval Medical University, Shanghai 200433, China; hydeng919@163.com (H.D.); xrshang0701@163.com (X.S.); 2 Department of Biochemistry and Molecular Biology, College of Basic Medical Sciences, Naval Medical University, Shanghai 200433, China; 3 College of Chemistry and Materials Science, Fujian Normal University, Fuzhou 350007, China; zhuhu@fjnu.edu.cn; 4 Key Laboratory of Biosafety Defense (Naval Medical University), Ministry of Education, Shanghai 200433, China

**Keywords:** saxitoxin (STX), paralytic shellfish toxins, voltage-gated sodium channels (NaVs), mechanism, treatment, detection

## Abstract

Saxitoxin (STX) is a potent toxin produced by marine dinoflagellates and freshwater or brackish water cyanobacteria, and is a member of the paralytic shellfish toxins (PSTs). As a highly specific blocker of voltage-gated sodium channels (NaVs), STX blocks sodium ion influx, thereby inhibiting nerve impulse transmission and leading to systemic physiological dysfunctions in the nervous, respiratory, cardiovascular, and digestive systems. Severe exposure can lead to paralysis, respiratory failure, and mortality. STX primarily enters the human body through the consumption of contaminated shellfish, posing a significant public health risk as the causative agent of paralytic shellfish poisoning (PSP). Beyond its acute toxicity, STX exerts cascading impacts on food safety, marine ecosystem integrity, and economic stability, particularly in regions affected by harmful algal blooms (HABs). Moreover, the complex molecular structure of STX—tricyclic skeleton and biguanide group—and its diverse analogs (more than 50 derivatives) have made it the focus of research on natural toxins. In this review, we traced the discovery history, chemical structure, molecular biosynthesis, biological enrichment mechanisms, and toxicological actions of STX. Moreover, we highlighted recent advancements in the potential for detection and treatment strategies of STX. By integrating multidisciplinary insights, this review aims to provide a holistic understanding of STX and to guide future research directions for its prevention, management, and potential applications.

## 1. Introduction

The ocean, as a rich reservoir of natural products, exhibits immense potential for drug research. Saxitoxin (STX) is a remarkable yet perilous neurotoxin, and as a pivotal component of the paralytic shellfish toxins (PSTs), STX is also one of the primary initiators of paralytic shellfish poisoning (PSP) [1,2,3], posing a substantial threat to both human health and the marine ecosystem.

STX emanates from a plethora of sources. Research indicates that STX can be produced both by dinoflagellates in marine environments and by cyanobacteria in freshwater environments. The former includes *Alexandrium* (comprising 10 species), *Pyrodinium bahamense*, *Gymnodinium catenatum*, and *Centrodinium punctatum*, which have long been considered the primary contributors to PSP [4,5,6,7,8]. Freshwater cyanobacteria include *Raphidiopsis brookii*, *Anabaena circinalis*, *Aphanizomenon* sp., *Raphidiopsis raciborskii*, and *Microseira wollei*, among others. It has been confirmed that 15 species of freshwater cyanobacteria possess the capability for STX synthesis [9,10,11,12,13]. Through the intricate food chain, STX accumulates in organisms such as shellfish and fish. Once humans consume these contaminated seafood items, they are at risk of intoxication. The symptoms of STX poisoning are diverse and severe, affecting the nervous system and including nausea, vomiting, oral paresthesia, visual disturbances, muscle paralysis, and myalgia. These symptoms may appear within 0.5 to 2 h after ingestion, and in severe cases, it can lead to respiratory paralysis and death [14,15]. Annually, there are approximately 2000 PSP cases worldwide, with a mortality rate as high as 15% in a comprehensive review [16], highlighting the urgent necessity for in-depth research on STX.

Internationally, the World Health Organization (WHO) and the European Food Safety Authority (EFSA) have set the limit for STX in 100 g of shellfish soft tissue at 80 μg [17]. The United States Environmental Protection Agency (EPA) has placed it on the candidate pollutant list 4 (CCL4) and list 5 (CCL5), and has established health advisories for drinking water regarding microcystin and cylindrospermopsin. Additionally, due to its high toxicity, stability, and potential use as a biological warfare agent, STX has received considerable attention in fields such as food safety, neuroscience, and ecotoxicology [18,19,20,21,22], and is also listed as a controlled substance under the Chemical Weapons Convention; related experiments are subject to strict safety approval.

Although numerous studies have demonstrated that the toxicity of STX is fundamentally attributed to its potent blockade of voltage-gated sodium channels (NaVs) as a specific site-1 sodium channel blocker (S1SCB) acting on the α-subunit [23,24], recent investigations have uncovered that β subunits may also exert a specific modulatory role in mediating the interaction between STX and sodium channels [25,26]. Moreover, different subtypes of NaVs in the human body exhibit varying binding capacities for STX [27,28,29]. Notably, apart from its primary action on NaVs, STX may also affect non-NaV targets to exert more complex toxic effects [30,31]. These aspects collectively constitute the intricate toxicity mechanism of STX. STX has a minimum lethal dose of 1–4 mg/kg and a median lethal dose (LD_50_) of merely 10 μg/kg (intraperitoneal injection in mice) [32,33], rendering it extremely toxic.

Over the years, extensive studies on STX have been undertaken. By systematically integrating the full-chain evidence spanning from “discovery” to “mechanism” and “application”, the present review innovatively constructs an interdisciplinary research framework, which comprehensively covers STX’s basic toxicology, biosynthesis pathways, and advanced detection technologies. It provides a systematic theoretical foundation for STX prevention and control, precise toxin monitoring, and rational drug design, thereby bridging the gap between fundamental research and practical applications in the field of marine toxins.

## 2. Historical Traceability and Structural Analysis

### 2.1. First Detection and Process

The discovery of STX can be traced back to 1957, when Hermann Sommer’s team first isolated the toxin from the Alaska butter clam (*Saxidomus gigantea*) and named it after its host [34]. In the early stages, research primarily focused on its toxic characteristics and found that it is closely related to PSP. The structural analysis of STX has been hindered due to the lack of high-purity samples and advanced characterization techniques.

In 1975, its chemical structure was completely analyzed; Schantz et al. further purified it and revealed its three-dimensional chemical structure through X-ray diffraction and nuclear magnetic resonance (NMR) techniques, confirming it as a tetrahydropurine derivative and unveiling its unique biguanide and tricyclic skeletal characteristics, while also affirming its role as the primary toxic component of PSP and its unique biguanide, tricyclic skeleton and hydrated ketone structure, laying the foundation for subsequent toxicity mechanism and synthesis research [35,36,37].

In 1977, the Tanino H. team successfully completed the total synthesis of STX racemate (d,l-STX) for the first time, marking the beginning of the artificial synthesis of STX [38]. Subsequently, the researchers developed various synthetic strategies for its complex triazole guanidine structure. For example, the Fleming team reported asymmetric synthesis methods in the 2000s, where natural STX possessed a specific optical purity (+)-configuration; the team achieved the stereoselective synthesis of (+)-STX through 17 reaction steps [39,40]. Iwamoto et al. optimized the synthesis route using the cyanation intermediate, which improved the yield [41,42]. These synthetic breakthroughs not only advanced the development of STX analogs (such as neoSTX and deoxynivalenol), but also provided tools for researching their structure–activity relationships.

### 2.2. Molecular Structure Characteristics

STX belongs to the class of pyrrolidine and purine alkaloids characterized by high polarity and hydrophilicity. Its molecular formula is C_10_H_17_N_7_O_4_ (molecular weight = 299.286 g/mol) and it has a CAS number of 35523-89-8. It belongs to pyrrolopurine alkaloids and tetrahydropurine derivatives, with its molecular framework consisting of a tricyclic fused system.

The active sites are concentrated in the guanidine groups at positions 7, 8, and 9, as well as in the C12 hydroxyl group. Additionally, while the formyl side chain is not a core active group, its spatial orientation can influence the binding specificity of the toxin to different subtypes of sodium channels (such as the differences between skeletal muscle and cardiac channels) [43,44,45]. Through reductive preparation, two stereoisomers can be derived: (12R)-dihydrosaxitoxin (12α-OH) and (12S)-dihydrosaxitoxin (12β-OH). The inactive dihydrosaxitoxin features a 12α-OH configuration, with its side-chain conformation resembling that of the ketone form. In contrast, the side-chain conformation of the 12β-OH isomer is similar to the hydrated form of the original toxin. Notably, the stereoelectronic effects of the 12β-OH group exert a significant influence on the side-chain conformation, which is critical for toxicity [46]. Notably, it has been shown that zetekitoxin AB (ZTX), as the only macrocyclic member of the STX family, has a C6-C11 isoxazolidine macrocyclic structure that is speculated to be the key to enhanced toxicity (LD_50_ is lower than STX). The macrocyclic backbone of ZTX enhances the binding affinity with NaVs by limiting the hydration conformation of C12 (ΔGhγ = 4.29 kcal/mol). This finding challenges the conventional wisdom that C12-hydrated ketones are necessary for the high toxicity of STX [47].

The STX pure product is a white crystalline solid, readily soluble in water, slightly soluble in methanol and ethanol, and insoluble in non-polar solvents. Analysis through the X-ray single-crystal diffraction of its aqueous solution crystallization reveals that the molecules form a strong hydrogen bond network through amino and sulfonic acid groups, and that the crystal structure contains ordered layers of water molecules [35,36]. It is readily soluble in water, sparingly soluble in methanol and ethanol, and insoluble in non-polar solvents. This solubility profile arises from the high polarity of the guanidino groups in the STX molecule, which facilitate the formation of extensive hydrogen-bonding networks, particularly in protic solvents; STX is thermally stable and exhibits high stability under acidic conditions (pH < 4) and low temperatures (−20 °C). It can be stored long-term in diluted hydrochloric acid without losing activity. Carbamate hydrolysis occurs only in highly concentrated acid solutions. Due to its heat and acid resistance, conventional cooking and processing cannot destroy STX, posing a significant threat to food hygiene and safety. However, it is prone to oxidation and inactivation in alkaline environments, with its toxicity rapidly diminishing [48,49]. Specifically, at a neutral pH, STX molecules exist in equilibrium in a divalent cationic hydrate, monovalent cationic hydrate, and monovalent ketone form. With the increase in pH, the C8 guanidine group donates hydronium ions, leading to a decrease in charge value, Additionally, the equilibrium between the ketone and hydrate forms affects the charge state of the molecule, potentially altering its interaction with sodium channels [46,50]. Saxitoxin with a deprotonated C8 guanidino group is susceptible to oxidation, so STX exhibits a more degradable characteristic when the pH value is >8 [51]. Furthermore, STX can be stored stably for a long time under light-proof conditions, while light exposure may accelerate its oxidative degradation. Solid STX remains stable in dry air, but is prone to oxidation when it absorbs moisture or is in solution. Therefore, it should be stored in a sealed and light-proof container [52].

The STX family includes various natural analogs. To date, 57 types of STX analogs have been described, all sharing a tetrahydropurine ring skeleton that can be substituted at positions C11, N1, and C13. Their structural differences primarily arise from the substitution or modification of functional groups. The saxitoxins themselves are further divided into classes such as C toxins, G toxins, and LW toxins depending on their chemical structures and substitutions [18]. They can be classified into several categories, such as non-sulfated, mono-sulfated, di-sulfated, and hydrophobic analogs, each with varying levels of toxicity [53,54]. The new stonehouse clam toxin (neo-STX), based on STX, has an added hydroxyl group at the N1 position, enhancing the hydrogen bond interaction with the sodium channel Tyr401, resulting in a higher affinity than STX [55]. In decarbamoylsaxitoxin (dc-STX), the C12 trancarbamyl group is missing, and the toxicity is reduced, but some channel inhibitory activity is retained [56]. Gonyautoxin (GTX) is generated through mono-sulfation at the N21 or O22 position of STX, which can then be di-sulfated to produce C-toxins [57]. The research on STX analogs is crucial for reducing PST toxicity and chemical or enzymatic degradation, in order to develop detoxification methods, while also exploring the unique medicinal potential of these toxins [58].

## 3. Molecular Biosynthesis

### 3.1. Biosynthetic Gene Clusters and Synthetic Pathways

The biosynthetic gene cluster of STX (Sxt gene cluster) was first identified in cyanobacteria, and genomic research showed that this gene cluster contained approximately 36 genes that encoded various functional enzymes, such as methyltransferases and aminotransferases [1].

**Figure 1:**
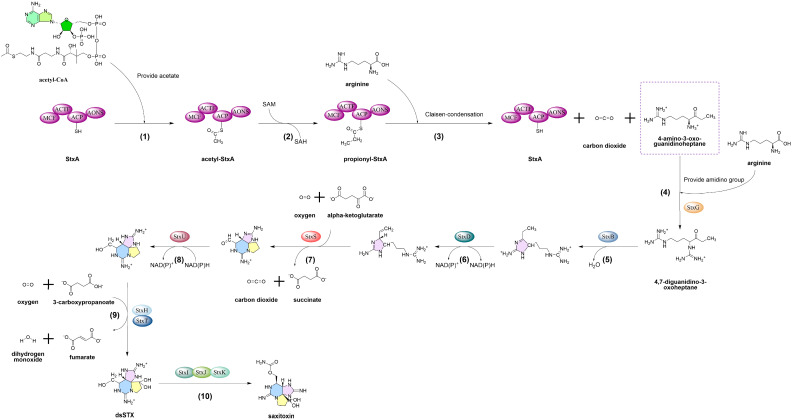
Biosynthesis pathway of STX [1,59]: Steps 1–3, Claisen condensation: Acetyl-CoA transfers an acetate group to the SxtA complex (subunits: MTF, ACP, ACTP, and AONS); methyl donation from SAM generates propionyl-SxtA, which undergoes Claisen condensation with arginine. Step 4, amidino group transfer: SxtG transfers an amidino group from arginine to the SxtA-derived intermediate, forming 4,7-diguanidino-3-oxoheptane. Steps 5–7, cyclization and double bond formation: Step 5, SxtB mediates cyclization of the amidino intermediate, forming the first heterocycle; Step 6, SxtD introduces a C1–C5 double bond; Step 7, SxtS catalyzes epoxidation of the new double bond, and epoxide ring opening generates aldehydes, triggering bicyclization. Steps 8–9, reduction and hydroxylation: Step 8, SxtU reduces the terminal aldehyde to an alcohol; Step9, SxtH/SxtT catalyze C12 dihydroxylation, with O_2_ and succinate as co-substrates. Step 10, carbamoyl transfer: the SxtI/SxtJ/SxtK complex transfers a carbamoyl group to the dihydroxylated intermediate (dsSTX), yielding saxitoxin (STX).

The biosynthesis of STX is a highly complex process (Figure 1), commencing with the polyketide synthase-like enzyme SxtA, and this enzyme contains multiple catalytic domains (*sxtA1*-*A4*). Among these, the *sxtA4* domain (functions as condensation domain) is considered a critical marker for toxin synthesis. SxtA is responsible for initiating the synthesis of the STX core structure using arginine and acetyl-coenzyme A (acetyl-CoA) as starting substrates. This step generates the intermediate product 4-amino-3-oxoadipate, which provides the structural framework for subsequent reactions. The subsequent step involves the participation of SxtG and SxtB in the guanylation reaction, catalyzed by guanylyltransferase, which introduces the guanidine derived from arginine into the molecular framework, forming the characteristic guanidine ring structure of the toxin. SxtS is a 2-oxoglutarate-dependent (2OG) dioxygenase that executes the consecutive epoxidation of new double bonds and opens the epoxide to form an aldehyde, accompanied by bicyclization. The dehydrogenase SxtU then reduces the terminal aldehyde of the STX precursor. Following this, SxtH and SxtT (the terminal monooxygenase subunit and the associated ring-hydroxylating dioxygenase encoded by the respective genes) catalyze the consecutive hydroxylation at C-12, resulting in dcSTX. Subsequently, SxtI combines with SxtJ and SxtK to catalyze the formation of STX, with the entire process encompassing approximately 30 biosynthetic steps [1,59] (Figure 1).

On the basis of STX, STX analogs are regenerated through specific reactions, and this process and the transport of toxins are involved in the transport of *sxtL*, *sxtN*, *sxtO*, *sxtR*, *sxtX*, *sxtW*, *sxtZ*, *sxtPER*, and other proteins encoded by the *sxtACT* gene [1,60,61].

The conservation analysis of the gene clusters indicates that the Sxt gene clusters of different toxin-producing cyanobacteria (such as *Raphidiopsis brookii* and *Cylindrospermopsis raciborskii*) have high homology, but that there are species-specific modifying enzymes that lead to the diversity of toxin analogs [62,63,64].

Additionally, research utilizing the *Alexandrium minutum* strain indicates a significant correlation between the toxin content of the strain and the expression of the fourth domain in the *sxtA* gene (*sxtA4*), as well as the presence of genetic subtypes. The quantitative polymerase chain reaction (qPCR) determination based on *sxtA4* has been applied in the study of toxic algal blooms associated with PST in marine environments, demonstrating broad application prospects [60,65]. There are also newly designed *sxtB* TaqMan probes for the targeted detection of the STX’s biosynthetic gene *sxtB* from *Alexandrium catenella* and *Alexandrium Pacificum* [66]. Notably, dinoflagellates, as the primary producers of STX, have posed challenges in identifying their biosynthetic genes. A recent comparative transcriptomic study of *Gymnodinium catenatum* identified over 1000 homologs of STX biosynthesis genes in this species. Among these, *sxtA* and *sxtG* exhibited significantly higher expression levels than other genes, suggesting their central roles in the toxin biosynthesis pathway. Intriguingly, the study revealed the absence of *sxtB*. This finding implies that *Gymnodinium catenatum* may employ alternative metabolic routes for STX biosynthesis or rely on functionally redundant genes to compensate for the loss of *sxtB* [67].

### 3.2. Environmental Regulation of Toxin Synthesis

The biosynthesis of STX is coordinately regulated by various environmental factors, including nutritional conditions, light, temperature, salinity, and others [68,69,70].

Different toxin-producing algae have their own suitable temperature ranges for growth and STX synthesis. Within the appropriate temperature range, enzymatic activity is high, and reactions related to the STX synthesis pathway can proceed smoothly. Extreme temperatures, whether too high or too low, can affect enzyme activity and thus inhibit STX synthesis. Additionally, temperature can influence the expression of core genes *sxtA* and *sxtG*, thereby affecting STX content, particularly evidenced by the increased expression of *sxtA* and *sxtG* under low-temperature stress [71].

The intensity and quality of light can affect the photosynthesis and related metabolic pathways of toxin-producing algae, thereby influencing the synthesis of STX [72,73]. Appropriate light intensity can promote the growth and metabolism of algae, facilitating the synthesis of STX, while excessive or insufficient light can inhibit STX production. Research indicated that an increase in temperature combined with adequate light can enhance the biomass of *Raphidiopsis raciborskii*. However, excessively high temperatures can lead to a decrease in its toxicity [74].

Toxic cyanobacteria (such as *Raphidiopsis raciborskii*) and dinoflagellates (such as the genus *Alexandrium*) perceive environmental changes and dynamically adjust the expression of the Sxt gene cluster to adapt to ecological pressures and maintain competitive advantages [75,76]. Specifically, changes in the nitrogen and phosphorus content in nutrients lead to nutritional stress, with nitrogen stress inhibiting the growth of *Alexandrium minutum* and reducing the biosynthesis of STX, while phosphorus deficiency can lead to an increase in the biosynthesis of saxitoxin [77,78]. The NO_3_^−^ concentration also affects the expression of *sxt* core genes and affects the production of STXs, suggesting that N is directly involved in the biosynthetic pathway of STXs [79].

Apart from the natural environment, the biological environment can also affect the content of STX. For instance, under the chemical induction of the zooplankton Daphnia gessneri, the *sxt* genes of *Raphidiopsis raciborskii* (Cyanobacteria) are upregulated [80]. Other studies have shown that *Jannaschia cystaugens* (representing *Alexandrium* cyst formation-promoting bacteria, Alex-CFPB) significantly influences the life cycle and toxin production of *Alexandrium pacificum*. Co-culturing with *Jannaschia cystaugens* induces stress that promotes the synthesis of highly toxic PST, leading to increased intracellular toxin levels, specifically involving oxidative stress, nutrient stress, and quorum sensing signals, all of which contribute to an increase in toxin synthesis [81].

### 3.3. Latest Research Developments

Although significant achievements have been made in the biosynthesis research of STX, many details still require refinement.

In exploring the details of the STX biosynthetic pathway, multi-omics technologies such as metabolomics and proteomics have played a crucial role. Through metabolomics, it is possible to comprehensively analyze the metabolite profiles of toxic algae at different growth stages and under various environmental conditions, and by utilizing proteomics, it is possible to identify differentially expressed proteins in toxic algae under varying environments, thus providing a pathway for the in-depth exploration of the dynamic changes in the STX biosynthetic pathway under environmental influences [2].

In addition, biotechnological techniques provide new strategies for the efficient production of STX, and researchers are attempting to reconstruct the STX biosynthetic pathway in heterologous hosts (such as *Escherichia coli* and *Synechocystis*) to achieve the extracellular synthesis of PST [82,83]. Jiao Y et al. established an asymmetric synthesis strategy featuring an intramolecular [2 + 2] photocycloaddition of an alkenylboronate ester equipped with a new chiral auxiliary, leveraging chiral additives to stabilize the transition state through hydrogen bonding, which achieved high stereoselectivity in constructing tricyclic frameworks and offered a novel paradigm for synthesizing complex toxins [84]. Furthermore, Greenhough H et al. scaled up the cultivation of *Alexandrium pacificum* using a 1250 L photobioreactor (PBR), thereby surpassing the production limitations of traditional natural sources for GTX-1,4. This advancement provides a gram-scale supply of toxins for preclinical research and offers a groundbreaking solution for the production of STX and its analogs [85].

## 4. Bioenrichment of STX: Pathways, Influencing Factors, and Ecological Impacts

The traditional route by which STX causes human poisoning involves the production of toxic dinoflagellates (such as the genus *Alexandrium*), their concentration through filter-feeding bivalves (mussels, oysters, and clams), and subsequent ingestion by humans, representing a common pathway in reported poisoning incidents. This pathway is closely associated with the species composition, geographic distribution, and toxin-producing capacity of the involved dinoflagellate populations. These factors are further influenced by environmental conditions, including salinity, temperature, and eutrophication levels [86,87,88].

Different traditional media have variations in the speed of toxin removal, retention time, and the retention of toxic components. The toxicity produced by dinoflagellate species also varies, resulting in different toxic effects on humans. Many countries have implemented monitoring programs for STX in toxic microalgae and shellfish, which help minimize public health risks [89].

Non-traditional media include non-bivalve invertebrates (gastropods and cephalopods), crustaceans, fish, annelid worms such as *Eudistylia* sp., and echinoderms like *Asterias amurensis*, *Astropecten scoparius*, *Astropecten polyacanthus*, and *Pisaster ochraceus*. Among these, although gastropods and cephalopods accumulate STX, they do not cause significant negative effects, while the yellow crab is the most commonly known non-filter-feeding non-molluscan species containing STXs. The mechanism by which STX is found in fish involves the transport of STXs through the food chain and the dissemination and accumulation of toxins through zooplankton [90,91]. Research indicates that the internal organs are the primary sites for PST accumulation. Simultaneously, these organs exhibit a higher STX clearance rate and excrete via the kidneys. Among STX and its analogs, those with higher solubility have a greater excretion rate [77,92]. The regulation and control of these non-traditional media is of significant importance for preventing STX poisoning incidents.

In the field of ecotoxicology, the accumulation of STX at the top of the food chain can lead to population cascading effects. For instance, high concentrations of STX in shellfish can cause foodborne poisoning, including PSP [93,94]. Toxins’ metabolic transformation significantly affects their toxicity level. The latest studies show that when mussels are exposed to dynamic algal cell densities (simulating natural blooms), the concentration of PSTs (paralytic shellfish toxins) in their bodies increases by 1.8 times compared with exposure to fixed densities. Moreover, low-toxicity sulfocarbamoyl toxins (such as GTX5) are converted into highly toxic derivatives (e.g., dcSTX) through decarbamoylation reactions, a process associated with the inhibition of sulfotransferase activity in the hepatopancreas [95]. The consumption of contaminated fish may lead to the mass death of marine predators that feed on fish (such as seabirds and cetaceans) [96,97,98]. Exposure to STX during the development of zebrafish can lead to changes in the expression of genes related to axon growth and affect the functionality of NaVs [99]. Studies on *Saxidomus gigantea* (Alaska butter clams) have shown that when humans consume 200 g of tissue containing 900 μg of saxitoxin (STX), the probability of severe symptoms in average males reaches 11%, and the mortality risk is 0.27%. This confirms the potential threats of STX to the ecological chain and human health [100].

The acute guideline value for STXs proposed by the WHO is 0.003 mg/L (3 μg/L) for the total STXs (including congeners, free forms, and cell-bound forms). Exposure routes include drinking contaminated surface water, consuming contaminated marine shellfish, and having contact with high-concentration water bodies during recreational activities. In terms of protection, avoiding contact and accidental ingestion is fundamental. So far, there is no specifically approved antidote, and treatment after poisoning mainly focuses on symptomatic and supportive care [101,102]. Health risks should be addressed through water source protection and water treatment process control, while paying attention to their impacts on the sensory quality of drinking water. Controlling water eutrophication can reduce the risk of cyanobacterial blooms. Additionally, water contaminated with STXs can be treated to mitigate poisoning risks. Extracellular STXs can be physically removed via activated carbon adsorption or membrane filtration [103]. Alternatively, chemical inactivation methods such as potassium permanganate, ozonation, and chlorination, etc., can disrupt the toxin structure. However, the risks of disinfection by-products should be noted [104,105]. In addition, H_2_O_2_ may not be able to effectively degrade saxitoxin [106]. Biological activity-based removal or degradation methods, such as adsorption by lactic acid bacteria and degradation by bacteria isolated from the digestive tract of blue mussels (*Mytilus edulis*), have been reported [107]. To remove intracellular toxins, complete cyanobacterial cells need to be eliminated, which can be achieved through traditional coagulation and sand filtration processes [108]. During the removal process, care must be taken to prevent cell lysis and the release of toxins, as the review [109] showed.

## 5. Toxicity Mechanisms of STX

STX, as a guanidine alkaloid, features a core structure with two guanidine groups, both belonging to the guanidinium toxin family with TTX. These guanidine groups exhibit an extremely high affinity with and ion flux blocking ability towards NaVs [25,110], which endows STX with high biological activity and extreme toxicity.

### 5.1. Blocking Mechanism of STX on NaVs

Toxins can be roughly divided into two categories: pore blockers that physically close the pore of the channel and “gating modifier toxins” (GMTs) that act on the voltage-sensing domain of the channel to cause abnormal activation (opening) or inactivation (closing) of the sodium ion channel.

Mammalian NaVs consist of a pore-forming α subunit and one or more β subunits. The α subunit is the main part that forms the ion channel pore and has voltage-sensing and gating functions [111,112]. The α subunit is folded from a single polypeptide chain containing four homologous repeat sequences. Each repeat sequence contains six transmembrane helix segments (S1–S6). These segments are connected by extracellular and intracellular loops. S1–S4 form the voltage sensor, and S5 (outer helix), S6 (inner helix), and the P loop that re-enters the extracellular membrane between S5 and S6 together form the pore domain [113,114]. Site 1 on the α subunit is the common receptor-binding site for these types of toxins, such as STX, tetrodotoxin (TTX), and polypeptide toxins like μ-conotoxin [25,115]. The active sites of STX are the guanidyl groups at positions 7, 8, and 9. They have a high affinity with the amino acid residues at site 1 of the voltage-gated Na⁺ channel on the excitable cell membrane, thereby physically blocking the pore [29]. Through the analysis of the structure of the sodium channel–STX complex via X-ray crystallography and cryo-electron microscopy, it was observed that after STX binding, the selective filter region of the channel contracted, narrowing the ion permeation path and preventing sodium ions from passing through smoothly [116].

**Figure 2:**
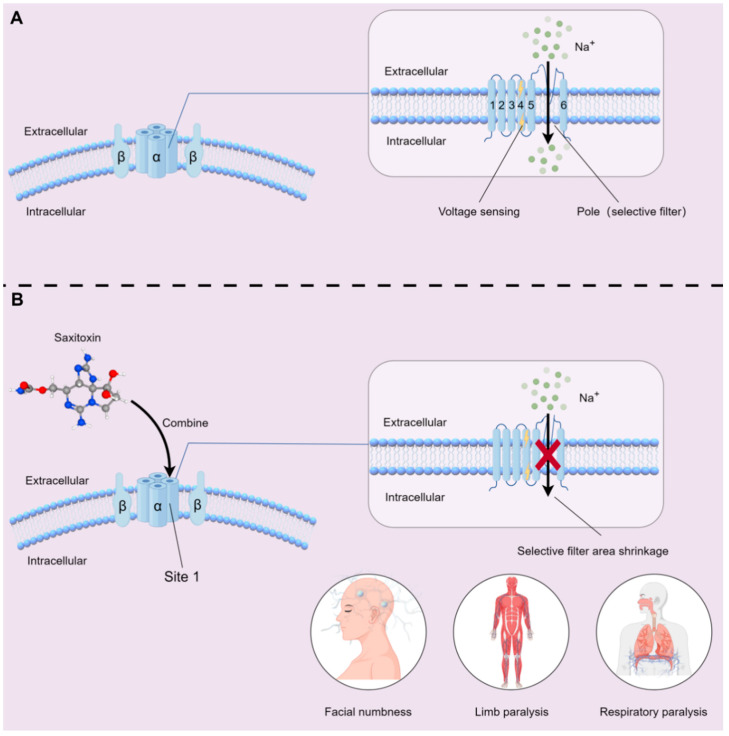
Schematic of STX Blockade Mechanism on NaVs: (**A**) Structural organization of NaV complex: The NaV complex is composed of a pore-forming α subunit and one or more β subunits [113]. The α subunit, a single polypeptide chain with four homologous repeat domains (I–IV), constitutes the ion-conducting pore and voltage-sensing apparatus. Each domain contains six transmembrane helices (S1–S6)—S1–S4 form the voltage sensor, while S5–S6 form the pore domain [114]; (**B**) STX binding and functional disruption: Upon binding to the outer vestibule of the α-subunit pore via site 1, STX induces conformational contraction of the selective filter, restricting Na⁺ influx [24]. This α-subunit-mediated disruption abrogates neuronal action potential generation (Vmax reduction ≥ 80%) and propagation, leading to clinical conditions: facial paresthesia, limb paralysis, and respiratory arrest, etc. [23,30]. (Some drawing elements are from www.figdraw.com).

STX blocks the influx of sodium ions, inhibiting the establishment and collapse of the transmembrane electrochemical charge gradient in animal nerve, muscle, and endocrine cells. This ion imbalance hinders the initiation of action potentials [117,118], leading to serious consequences. The symptoms of STX poisoning usually appear within 30 min after ingestion, including oral numbness, limb paralysis, and breathing difficulties, etc. In severe cases, it can cause death within a few hours [119,120] (Figure 2).

### 5.2. Association of β Subunits in the Action of STX

Furthermore, the process of STX blocking sodium channels also involves interactions with β subunits. The β subunit is not just a simple accessory to the pore-forming α subunit. Under normal circumstances, β subunits play an important role in the localization of sodium channels, function regulation, and coupling with other cell signaling pathways [115,121], allowing for the precise control of excitability in a cell type-specific manner. At the same time, the regulatory effect of the β subunit on voltage-gated potassium channels affects neuronal excitability. Abnormal neuronal excitability is related to many nervous system diseases. Therefore, this regulatory effect may play a role in the occurrence and development of nervous system-related diseases [26].

Messner, D.J. and Catterall, W.A. detected the functional integrity of the resulting protein complexes by selectively removing β1 or β2 subunits from the detergent-solubilized channels in rat brains. They found that selectively removing the β1 subunit from the detergent-solubilized channels in rat brains led to a complete loss of 3H-labeled STX binding activity in sodium channels, confirming that the presence of the β1 subunit is crucial for maintaining the high-affinity conformation of the STX binding site on sodium channels [122].

Nonetheless, currently, there are very few studies on the specific role of β subunits in the process of STX toxicity, and further exploration is needed.

### 5.3. Affinity Difference in Different Subtypes of NaVs to STX

In the human body, there are nine subtypes of sodium channels, such as Nav1.1–Nav1.9. They are specifically distributed in different tissues. The amino acid sequences, spatial conformations, and accessory subunits of different subtypes of sodium channels vary [27,28,123], resulting in different binding abilities of STX to each subtype.

However, STX binds to the amino acid residues at site 1, which is located on the outer surface of the excitable cell membrane and closer to the outer opening of the sodium channel [29,43]. Sodium channels in nerve tissues have a high affinity for STX. Ritchie and Rogart conducted binding experiments using radioactively labeled STX with rabbit vagus nerves, lobster walking leg nerves, and gar olfactory nerve fibers and found that STX can tightly bind to the NaVs on nerve cell membranes and effectively block nerve impulse conduction at extremely low concentrations [124].

Nav1.4 is mainly distributed in skeletal muscle. The high affinity of Nav1.4 in skeletal muscle for STX may be determined by the charge complementarity of acidic residues in the pore region, the cooperative effect of domains, and subtype-specific conformations [113,125], which provides a key molecular target for the development of toxin antagonists targeting skeletal muscle sodium channels. In contrast, the sodium channels in cardiac muscle tissue have a relatively low affinity for STX [126]. Catterall and Coopersmith used radioactive ligand binding experiments to detect STX receptor sites in vertebrate heart tissues. However, the binding ability of heart tissues to STX is weaker than that of nerve tissues, and a higher concentration of STX is required to produce a significant inhibitory effect [127].

### 5.4. Potential Toxicity Targets of STX

Interestingly, although the toxicity of STX mainly stems from the inhibition of specific NaVs, STX can also affect other channels, enzymes, and proteins, thereby exerting toxic effects [128]. Studies have found that STX also affects L-type calcium channels [30] and HERG potassium channels [31], interfering with the ion balance and electrophysiological activities within cells. In addition, STX may also act on neuronal nitric oxide synthase, STX-metabolizing enzymes, a transferrin-like family of proteins, and a unique protein found in the blood of pufferfish. Although the specific mechanisms and degrees of influence remain unclear, these additional effects may further exacerbate the toxic effects of STX on organisms [120,128].

### 5.5. Synergistic Effect of STX with TTX

In addition to structural analogs, the combined exposure effects of STX with other toxins have garnered increasing attention in recent years. Despite their pronounced chemical, structural, and affinity disparities, both STX and TTX are categorized as S1SCBs, and their neurotoxicity may exhibit additive effects [129,130].

Furthermore, investigations into the combined toxic effects of STX and TTX have made significant strides recently. Concurrently, Andrea Boente-Juncal et al. validated through chronic toxicity experiments that combined exposure to TTX and STX elicits additive toxic effects, particularly when the STX dosage exceeds the maximum exposure threshold recommended by the EFSA, leading to significantly elevated mortality and biochemical derangements. They demonstrated via intragastric gavage that co-exposure to STX and TTX in mice induced significantly more aberrant changes in blood biochemical parameters and higher mortality rates compared to single-toxin exposure groups, thereby confirming their combined toxic effects [131]. Nevertheless, the intragastric gavage methodology harbors methodological limitations, as it may lead to inadvertent pulmonary aspiration of the administered toxins—potentially causing rapid lethality—and mechanical reflux induced by gastric tube insertion [132,133]. Nevertheless, Finch et al. systematically demonstrated the additive toxicity of STX and TTX through LD_50_ determinations via multiple routes (intraperitoneal injection and feeding), a comparison of predictive models with experimental data, and consistency analysis of action mechanisms and clinical symptoms [134]. Despite the lack of in-depth explanations of the mechanisms of the combined effects of toxins, these findings provide a scientific basis for incorporating TTX into the regulatory system of PSTs.

## 6. Self-Resistance Mechanisms to STX Enlighten Treatment Development

Currently, there is no specific antidote for STX poisoning. Treatment mainly involves symptomatic supportive therapies such as artificial ventilation and gastric lavage, as the review summarized [135]. However, research on the strategies of organisms in nature to cope with STX exposure has inspired the research on STX treatment [136,137]. In recent years, relevant research achievements in exploring STX antagonists and optimizing toxin delivery systems have also provided clues for the development of drug treatments.

### 6.1. Target Protein Resistance Mutations

Target protein resistance mutations play a crucial role in the research on the mechanisms of biological toxicity resistance and have been investigated in depth and systematically [137,138]. Soft-shell clams (*Mya arenaria*) from areas affected by “red tides” have been found to be more resistant to PSTs. This is due to a natural mutation of a single amino acid residue, which reduces the affinity of the STX binding site in the sodium channel pore of resistant soft-shell clams (but not sensitive ones) to one-thousandth of the original level [139]. When cysteine (Cys) at position 374 of the cardiac sodium channel α subunit (RHI) is replaced by tyrosine (Tyr) (similar to the situation of the μl subtype), the sensitivity of RHI to STX changes significantly [140], which may significantly affect the STX resistance of organisms. When single-gene mutations were studied in the SS2 and adjacent regions of the four internal repeat sequences in sodium channel II, it was found that mutations involving a negatively charged residue clustered at specific positions significantly changed the STX sensitivity, and that these mutations may affect the extracellular mouth and/or pore wall structure of the sodium channel [125]. All these mutations have an impact on the STX resistance of organisms.

### 6.2. Toxin Sequestration

Soluble STX-binding proteins found in some organisms such as frogs, pufferfish, cockles, and crabs constitute a special mechanism for organisms to cope with toxins—toxin sequestration [141]. Among the strategies of organisms to cope with toxins, the resistance mechanism of frogs to STX has attracted much attention [142]. It has been found that saxiphilin (Sxph) in bullfrogs can bind to STX with a high affinity (K_d_ is approximately 0.2 nM) [143,144,145]. In the study of the binding site of STX to NaVs, it was found that Sxph and Navs use similar molecular logic to recognize STX. Both use side-chain carboxylates to interact with the five-membered and six-membered guanidyl groups of STX. Moreover, Sxph can bind STX through a specific binding pocket in the C1 domain, thus acting as a high-affinity “toxin sponge” protein to prevent STX poisoning. In addition, a similar sequence of Sxph, SxphNP, was found in the Himalayan frog (Nanorana parkeri), which also has the key characteristics of recognizing STX [128,146]. These findings expand the research direction of developing effective STX antidotes based on biological resistance.

### 6.3. Biotransformation and Metabolism

Some reports have indicated that the digestive glands of bivalve molluscs possess a high capacity for PST transformation [147,148], suggesting the presence of microorganisms and/or toxin-transforming enzymes within them, as demonstrated in the review [149]. Vasama Mshou et al. were the first to confirm that *Lactobacillus rhamnosus GG* and *LC-705* strains (both in viable and non-viable forms) can remove STX and neoSTX from neutral solutions with a pH of 7.3 and acidic solutions with a pH of 2, achieving removal rates of as high as 77–97.2% [150]. *Pseudoalteromonas* bacteria are capable of degrading PSTs, and STX is one of them. These bacteria have the potential to remove STX. Such bacteria are relatively common in the marine environment. They may degrade toxins through special enzymes secreted by themselves or metabolic pathways. Marine bacterial isolates cultured from the digestive tracts of blue mussels (*Mytilus edulis*) contaminated with PST can reduce the overall toxicity of algal extracts to less than 10% of the original within 3 days [151]. These bacteria and similar ones may assist marine bivalves in the natural metabolism and elimination of PSTs and other marine biotoxins, and also promote research on the biological treatment of STX poisoning.

Meanwhile, some newly reported C-11 hydroxyl analogs of PSTs in shellfish are considered novel derivatives of PSTs and are called “M-toxins” [152,153,154,155]. The research team led by Li Aifeng confirmed that the lower the pH and temperature under experimental conditions, the more stable the M-toxins. Compared with common components of PSTs, the stability of M-toxins is poor. They also confirmed the chemical transformation pathways of “M-toxins” as M1→M3→M5, M2→M4→M6, and NEO→M10 [156]. This is of great significance for understanding the detoxification mechanism of shellfish and achieving rapid detoxification by converting highly toxic PSTs into weakly toxic M-toxins. It also provides new ideas for studying the detoxification mechanism of organisms to STX.

## 7. Detection

In the context of the high risk of STX, countries around the world have listed it as a mandatory item for aquatic product safety inspection, and the detection technology of STX and its derivatives is the key to its bioaccumulation and toxicological mechanism study. At present, there are various detection methods, mainly including biological detection methods and instrument detection methods, etc.

Biological detection methods, based on the biological activity of toxins, were the main means for early STX detection. The Mouse Bioassay (MBA) is a classic method for early STX detection. It involves the intraperitoneal injection of sample extracts and semi-quantitative analysis of STX toxicity based on mouse death time. Since it was confirmed as the statutory detection method by the Association of Official Analytical Chemists (AOAC) in 1958 (AOAC 959.08), it has been used as the reference standard for other detection techniques [157]. However, it has limitations such as an inability to distinguish toxin analogs, low sensitivity, and animal ethics controversies [158]. Additionally, the Daphnia acute toxicity test utilizes the sensitivity of *Daphnia pulex* (limit of detection (LOD): 0.3–10 ng equivalent to saxitoxin (Eq.STX)/L) and *Moina micrura* (LOD: 2.7–10.7 ng Eq.STX/L) to STXs, serving as a rapid screening tool for environmental samples [159]. Similarly, the speckled cockroach (*Nauphoeta cinerea*) bioassay can act as a supplementary field screening tool for STXs, with its core value lying in cost and speed advantages, though specificity remains low [160].

Cell-level detection, achieved through toxicity effects mediated by NaVs, can replace traditional mouse bioassays. For example, the Neuro-2a cell-based assay operates on the principle that veratridine, in the presence of ouabain, increases sodium influx in the mouse neuroblastoma cell line Neuro-2a (ATCC, CCL131), leading to cell swelling and death [159]. STX specifically blocks NaVs, antagonizing the toxic effect of veratridine. The toxicity of STX is indirectly reflected by detecting functional changes in intracellular ion channels or cytotoxic effects (such as MTT metabolism and lactate dehydrogenase release). Similarly, this method can also be used for the detection of sodium channel-blocking toxins such as TTX [161,162]. However, this method requires a long analysis time. The analysis time can be shortened by using modified forms of short toxins or the calcium agonist maitotoxin [163,164]. In addition, it also has high requirements for reagents, professional knowledge, and equipment.

With the acquisition of anti-STX antibodies, immunoassay techniques have been developed. The enzyme-linked immunosorbent assay (ELISA) method is the most commonly used, relying on the color reaction between enzyme-labeled antibodies and substrates for detection, including direct and indirect ELISA [165,166]. Immunoassay can achieve the rapid screening of PSP, but its cross-reactivity to different toxins is low, making it difficult to fully reflect the composition of toxic components in samples. Therefore, when detecting STX and its analogs, additional approaches are required to identify and quantify specific toxins, which highly depends on laboratory equipment [165]. The subsequently developed MIST Alert™ rapid detection method can be used for the determination of the total PSP toxicity and related toxin toxicity in acidic crude toxin extracts. It achieves a 100% detection rate for toxic extracts containing at least 80 μg Eq.STX/100 g, with a LOD of approximately 40 μg Eq.STX/100 g (varying with toxin profiles). This method features high sensitivity, rapid detection, and does not require specialized tissue culture equipment or expertise. However, it is unable to distinguish individual toxin components [167,168]. Rabbit monoclonal antibodies (r-mAbs) developed by Li et al. achieved the ultra-sensitive recognition of STX through a single-cell sorting and cross-screening strategy. The sensitivity is 100 times higher than that of traditional mouse antibodies, which solves the problem of the poor stability of traditional antibodies in complex matrices [169].

The receptor binding assay (AOAC 2011.27) utilizes the principle that PSP toxins can competitively bind to NaVs with [3H]-STX diHCl. This method applies isotope labeling to detect PSP toxins, featuring rapidity, reliability, and accuracy [170]. The substitution of Na+ channel proteins with saxiphilin, which specifically binds to STX, has been studied to be specific for STX identification and can be distinguished from TTX identification [171,172].

In chemical instrumental analysis, high-performance liquid chromatography (HPLC) was the first instrumental analysis technique applied to the detection of PSTs. It offers advantages such as high sensitivity, strong specificity, a low LOD (0.1 μg/kg), and fast analysis speed. HPLC can simultaneously provide qualitative and quantitative information for multiple toxins, making it the core method for toxin detection currently. Due to the weak chromophoric group of STX, pre-column oxidation (AOAC 2005.06) or post-column oxidation (AOAC 2011.02) is required before detection to generate fluorescent chromophores (e.g., dimethylaminobenzoic acid derivatives), which are then detected using a fluorescence detector. Among them, post-column oxidation HPLC is noted for its operational simplicity, strong matrix tolerance, and high stability [173,174].

Subsequently, with the advancement of mass spectrometry technology, the combination of liquid chromatography and mass spectrometry (LC/MS) has emerged as one of the preferred analytical methods for the highly selective and sensitive quantification of toxins. In a study, dansyl chloride was used for the chemical derivatization of STX, followed by separation via ultra-high-performance liquid chromatography (UHPLC) coupled with heated electrospray ionization (HESI), and quantification using a Q-Exactive mass spectrometer. This approach achieved a LOD as low as 0.01 μg/L [175,176]. LC-MS/MS serves as the gold standard for detecting guanidine toxins. Its chromatographic separation module is compatible with both HPLC and UHPLC systems [177]. Liquid chromatography separates STX via a chromatographic column by leveraging the partitioning difference between the stationary phase and mobile phase. Meanwhile, mass spectrometry ionizes STX through techniques such as electrospray ionization (ESI), generates characteristic product ions via collision-induced dissociation (CID), and enables the simultaneous qualitative and quantitative analysis of STX and more than 50 of its analogs using the multiple reaction monitoring (MRM) mode [178]. Subsequent improvements in the separation and extraction methods for STX have been made. For example, the combination of isotope dilution liquid chromatography tandem mass spectrometry (HILIC) and the isotope dilution method has enabled the simultaneous detection of multiple toxins and improved sensitivity [179,180]. The extraction and purification of STX are performed using immunoaffinity column (IAC) combined with solid-phase extraction (SPE) technology. STX is extracted with phosphate-buffered saline (PBS), separated on a TSK-GEL Amide column, and detected via LC-MS/MS. This method completely eliminates the matrix effects and does not require matrix matching, meeting the requirements for the detection of trace STX in bivalve aquatic products [181]. Additionally, extraction is performed by acidifying plasma samples and precipitating with acetonitrile, followed by LC-MS-MS analysis. The entire process takes ≤30 min, with a LOD of 2.8 ng/mL and a lower limit of quantitation (LOQ) of 5.0 ng/mL [182]. This technique utilizes the characteristic fragmentation of the guanidino group in STX molecules, combined with the high sensitivity of electrospray ionization (ESI) to distinguish structurally similar toxins (such as neoSTX and dcSTX) and achieve accurate confirmation in complex matrices (e.g., biological tissues and seawater). For instance, Dahlmann et al. used LC-ESI-MS/MS to simultaneously detect STX and microcystins in phytoplankton, confirming the tricyclic skeleton structure of STX through MS/MS fragment analysis [183]. LC-MS/MS offers significant advantages in STX detection, enabling simultaneous multi-component analysis without the need for derivatization, thus ensuring high operational efficiency. However, it also has limitations, such as expensive instrumentation and complex maintenance.

Thin-layer chromatography (TLC) is one of the traditional methods for detecting PSP toxins, which is currently used less frequently [184]. Research has been conducted on the chromatographic column for the separation of STX and its congeners using thin-layer chromatography in a rod shape, with detection by a flame thermionic detector (FTID). The detection limit for STX was 5 ng, while the detection limits for the other congeners also reached the ng level [185]. In addition, traditional techniques for detecting STX toxins include gas chromatography, ion-exchange column chromatography, and electrophoresis [184,186,187,188]. However, these traditional methods are currently less used due to their low resolution and poor sensitivity.

In recent years, new detection technologies have been continuously emerging. For example, some research is dedicated to developing detection methods based on biosensors, utilizing the specific interactions between biological recognition elements and STX, achieving the rapid and sensitive detection of STX through signal transduction, thereby enhancing selectivity and sensitivity [189,190]. These methods hold promise for overcoming some of the limitations of the traditional techniques, such as expensive instrumentation and complex operation, featuring advantages of portability, rapidity, high sensitivity, and low cost. They provide new possibilities for on-site detection and real-time monitoring. However, they also suffer from limitations, including complicated fabrication, short operational lifespan, complex manipulation, high professional requirements, and significant susceptibility to matrix effects [191,192,193]. In addition, microfluidic chip technology has also begun to be applied in the field of STX detection. The microfluidic chip can integrate multiple steps such as sample processing, reaction, and detection onto a small chip, offering advantages such as fast analysis speed, the low consumption of samples and reagents, and the ability to achieve high-throughput detection, thereby providing direction for the miniaturization, automation, and integration of STX detection. However, its fabrication is complicated, and each detection causes the consumption of the detection chip, resulting in a short operational lifespan [194,195].

Based on the existing research methods and their characteristics, combined with the scenario requirements for STX detection (on-site screening, laboratory confirmation, and research-grade analysis), the following feasible detection system of “rapid screening–precise quantification–mechanism verification” is proposed. By integrating the sensitivity, specificity, and operational complexity of different techniques, this system enables efficient detection. Quality control is essential before and during the detection process. The first stage involves on-site rapid screening, applicable to critical scenarios including aquatic product harvesting sites, the emergency monitoring of environmental water bodies, and initial food safety screening. The Daphnia acute toxicity bioassay and MIST Alert™ rapid test are employed here, leveraging their combined advantages of operational simplicity, cost-effectiveness, and rapid response. These techniques enable the preliminary triage of non-toxic samples, thereby streamlining the subsequent analytical workload. The second stage is the laboratory confirmation and quantification stage (applicable scenarios: regulatory sample re-inspection, market access testing, and acquisition of basic data for toxicological research). Firstly, the indirect ELISA method is adopted, which uses the specific binding of enzyme-labeled antibodies to STX for color development, rapidly quantifies the total STX equivalents in samples, and screens positive samples. The second step is precise analysis via HPLC (post-column oxidation method) or LC-MS/MS, with LC-MS/MS being the preferred choice. The third stage is the research-grade analysis and mechanism verification stage (applicable scenarios: STX toxicological mechanism research, identification of novel toxin analogs, and drug development target screening), involving the utilization of cell-based assays (Neuro-2a cell-based assay) and receptor-binding assays. By integrating LC-MS/MS for structural characterization with cell function analysis, this stage deepens STX research from the multi-dimensional perspectives of “toxicity effect–molecular mechanism–structural characterization” (Table 1).

## 8. Conclusions

STX, as a potent neurotoxin derived from marine dinoflagellates and freshwater or brackish water cyanobacteria, has been the subject of extensive research in recent years. This review has comprehensively summarized the current state of knowledge regarding STX, encompassing its discovery history, chemical structure, molecular biosynthesis, bioenrichment, toxicity mechanisms, self-resistance mechanisms, and detection methods.

The discovery and structural elucidation of STX were pivotal milestones in understanding its properties and biological activities. The identification of its unique tricyclic skeleton and biguanide group provided a foundation for subsequent studies on its interactions with biological targets, particularly NaVs.

In the field of toxicity mechanism research, the traditional mechanism of STX as a specific S1SCB of NaVs has been systematically elucidated. However, recent studies have continuously broken through the boundaries of cognition, not only discovering potential novel action targets of STX beyond NaVs, but also achieving key advancements in the exploration of biological natural resistance mechanisms. These innovative discoveries provide entirely new ideas and theoretical support for the development of STX-related drugs. Scholars such as Mingxuan Kai et al. have developed Neuron-MOF/SxtN-NPs (neuronal membrane-coated MOF nanoparticles loaded with SxtN enzyme), a dual-mode cellular nanoparticle formulation. They enable broad-spectrum neurotoxin neutralization via membrane mimicry and continuous detoxification through enzymatic activity. In STX-intoxicated mice, the nanoparticles show enhanced survival, no acute toxicity, and validate a biomimetic nanomedicine platform for neurotoxin countermeasures [199]. Dali Wang et al. have pioneered the design of an aptamer-based small-molecule therapeutic sustained-release system, applying STX to nerve blocks [200], which has inspired the progress of “seeking drugs from the ocean”.

As a highly toxic marine neurotoxin, STX as a single component has posed a threat to the marine ecological balance and human health. In natural environments, STX often forms complex contamination systems with multiple toxicants, and more than 50 natural structural analogs of STX have been identified. In this context, research on the combined action of toxins is gradually becoming a frontier in the field of STX toxicology. This research not only helps to reveal the real toxic risks of natural contamination systems, but also provides key scientific evidence for establishing the safety thresholds of marine toxins based on mixture effects and developing multi-target detoxification strategies.

In terms of detection technology, although numerous methods have been developed and applied to STX detection, and LC-MS/MS has become the most reliable method for detecting STX, systematically comparing the advantages and disadvantages of the existing detection methods and scientifically classifying their applicable scenarios still holds significant practical significance for achieving the rapid and precise detection of STX.

## Figures and Tables

**Table 1:** Established detection system for saxitoxin (STX).

Stage	Technical Method	LOD *	Application Scenarios	Key Features	Ref
On-site rapid screening	Daphnia acute toxicity bioassay	0.3–10.7 ng Eq.STX/L	Aquatic product preliminary screening	**Low cost and simple operation**	[158]
	MIST Alert™ rapid test	40 μg Eq.STX/100 g	Aquatic product preliminary screening	**Equipment-free and adapted for on-site rapid turnaround**	[168]
Laboratory confirmation and quantification	ELISA	0.015 ng/mL	Positive sample screening and preliminary quantification	**High stability and applicable for rapid triage of large-scale samples**	[196]
	HPLC (post-column oxidation method)	0.1 μg/kg	Routine sample quantification	**High specificity**	[181]
	LC-MS/MS	0.1 μg/kg	Complex matrices and multitoxin simultaneous analysis	**High specificity and capable of structural confirmation**	[178]
Research-grade analysis and mechanism verification	Neuro-2a cell-based assay	2 ng/L	Toxicological mechanisms and low-dose effect studies	Direct correlation between STX ion channel blockade toxicity and biological effect simulation	[197]
	Receptor-binding assay	2 ug Eq.STX/L	Toxin–receptor interaction mechanism	**Real-time reflection of binding kinetics and high sensitivity**	[198]

*: The limit of detection (LOD) may be affected by various factors such as experimental conditions, instrument performance, and sample matrix, which may vary in actual detection.

## Data Availability

No new data were generated in this review.
